# Assessments of epidemic spread in aquaculture: comparing different scenarios of infectious bacteria incursion through spatiotemporal hybrid modeling

**DOI:** 10.3389/fvets.2023.1205506

**Published:** 2023-09-13

**Authors:** HyeongJin Roh, Dhamotharan Kannimuthu

**Affiliations:** Pathogen Transmission and Disease Research Group, Institute of Marine Research, Bergen, Norway

**Keywords:** fish epidemiology, disease transmission, spatiotemporal hybrid simulation, compartment model, disease control in aquaculture

## Abstract

The sustainable development of the aquaculture sector is at risk due to the significant challenges posed by many emerging infectious diseases. While disease prevention and control measures are becoming increasingly critical, there is a dearth of studies on the epidemiological aspects of disease transmission in aquatic ecosystems. This study aims to forecast the spread of a bacterial disease between fish farms in two regions, Romsdalsfjord in Norway and Gujwa in South Korea by applying a DTU-DADS-Aqua spatiotemporal hybrid simulation model. The simulation model assessed the pattern of disease transmission between fish farms under different degrees of transmission power based on the distance between farms (*ScalingInf*), host susceptibility (*RelSusceptibility*), the origin site of disease, and the capacity of culling fish. The distance between fish farms was found to have significant associations with disease transmission. In most simulation conditions, the disease transmission between different bay management areas (BMAs) was not evident in Romsdalsfjord. In the Guwja region, where there are relatively narrow distances between fish farms, the spread of infectious disease was greatly affected by *ScalingInf*. The impact of *RelSusceptibility* on disease transmission patterns is a critical factor to consider in simulation modeling. When *RelSusceptibility* ranges from 0.5–1, there is little impact on the likelihood of disease transmission. Conversely, lower ranges (0.2 and 0.05) of *RelSusceptibility* result in a significant decrease in the area affected by the spread of disease. Eradication measures could control the patterns of infectious disease transmission, but the effectiveness of the depopulation strategy can be dramatically changed depending on the geographical environment. In conclusion, through a comparative analysis of the disease transmission and management scenarios, this study demonstrates the potential use of existing simulation models in predicting the spread of infectious diseases under different epidemiological circumstances and quarantine actions.

## 1. Introduction

Although the types of bacteria that are problematic for the aquaculture industry may vary depending on the region or country, farming system, and candidate host species, there is no doubt that many countries are facing the issues of bacterial diseases in aquaculture (1, 2). Despite the commercial availability of relatively robust vaccines for several bacterial diseases, efficacy issues persist due to various factors, such as differences in bacterial species and serotypes, mutations, and host factors (3). According to a Norwegian fish health report in 2021, an increase in the occurrence of bacterial diseases such as tenacibaculosis, pasterurellosis, and yersiniosis is reported in farmed salmon. Norwegian farmed salmon have been vaccinated against *Moritella viscosa*, the causative agent of winter-ulcer disease, but *M. viscosa* is still ranked as the second or third-highest problem in seawater aquaculture (4). This drives the use of antibiotics and other alternative methods for treatment, but their efficacy is inconsistent.

Bacterial diseases and related treatment issues in aquaculture are not confined to a single country. *Streptococcus parauberis* is a major bacterial pathogen causing continuous mortality and economic losses in olive flounder (*Paralichthys olivaceus*), which accounts for one of the biggest productions in farmed fish in Korea (5–8). Although several methods to treat and prevent Streptococcosis using commercial vaccines and antibiotics are available, the mortality has been known to be around ~15.8% in Korea (5, 9). It is hypothesized that *S. parauberis* is transmitted between adjacent fish farms through seawater. Indeed, Roh et al. (10) observed high numbers of *S. parauberis* using qPCR and the direct plating method (1–4 × 10^7^ copies 100 mL^−1^ or 2–7 CFU 100 mL^−1^) in the water off the Gujwa and Pyoseon regions of Jeju Island, where olive flounder is cultured the most in Korea. Considering that these bacterial pathogens can persist in a viable but nonculturable (VBNC) state in seawater and nutrient-deficient environments for extended periods, there has been a significant risk of disease transmission to neighboring fish farms over time (11, 12). However, the spread and extent of the disease within the local area under dimensional epidemiological factors, such as environmental conditions, the proximity of fish farms, the pathogen transmission potential, and host susceptibility, remain largely unknown. The absence of such information can lead to unfavorable outcomes in improving the fish farm environments from an epidemiological perspective. To address this knowledge gap, simulations considering multiple variables, including varying degrees of surveillance and quarantine policies, can predict best- and worst-case scenarios in the specific region where fish are intensively cultured. These simulations can inform appropriate action and management to improve the current situation.

In general, the calculation of basic reproduction number (R0 or R naught) and compartment models are widely used for epidemiological analysis to forecast infectious disease transmission in a specific closed population or herd (13–15). The basic reproduction number, defined as “the number of secondary cases which one case would produce in a completely susceptible population” by Dietz (13), is one of the simplest ways to predict whether a specific infectious disease in a population will be more prevalent (pandemic R0 > 1), have a minor change in transmission (endemic R0 ≈ 1), or diminish over time (R < 1) (16). However, R0 has limitations in simulating the disease prevalence in a specific population in real time because many epidemiologically important variances, such as host susceptibility to disease and the appearance of an individual obtaining immunity over time, have not been considered (17). On the other hand, compartmental models provide a range of options for categorizing individuals into stages such as susceptible (S), infectious (I), recovered/removed (R), and exposed (E). These models incorporate the progression of infection over time and include several optimized variants like SIS, SIR, SEIR, and SIRS, which can be adapted to align with the attributes of both the pathogen and the host (15, 18). Nevertheless, the compartment model faces significant challenges in simulating the disease spread across multiple herds concerning geographic characteristics. In order to overcome these limitations, several frameworks have been developed for predicting disease outbreaks and transmission. However, these frameworks differ in their underlying principles and the contextual factors that they emphasize [e.g. (19–24)]. In addition, most simulation models have been developed for terrestrial rather than aquatic animals, which can be problematic when they are directly used in aquaculture sites due to the big environmental differences. Recently, Romero et al. (23, 24) developed a stochastic, spatiotemporal hybrid simulation model (DTU-DADS-Aqua) modified from DTU-DADS-ASF. Its framework can trace the waterborne spread of infectious pathogens between fish farms or even net pens, to which the compartmental model and agent-based model are applied by considering farm-site hyperconnectivity based on the distance. The DTU-DADS-Aqua model is based on assumptions that the infectious pathogens are horizontally transmitted through the connectivity of seaway distance in the sea, fiords, rivers, and lakes under dozens of parameters relevant to pathogen factors, host factors, and the anthropogenic disease control actions (e.g., disease surveillance, detection, and depopulation) (23, 24). This study utilizes the DTU-DADS-Aqua tool to investigate the spread of potential bacterial diseases under a lower transmission power range following the distance between fish farms than viral diseases. The primary objective is to predict and simulate disease spread considering variables such as host susceptibility, site of the first outbreak, and intensity of quarantine actions in two distinct fish farming locations: Romsdalsfjord in Norway and Gujwa in South Korea. Different transmission scenarios were employed to simulate disease transmission, and the spatiotemporal hybrid simulation model was used to assess the impact of different proportions of immunized fish (0, 50, 80, and 95%) on the severity of emerging diseases in each closed fish farm.

## 2. Materials and methods

### 2.1. Fish farm and simulation area

**Figure 1:**
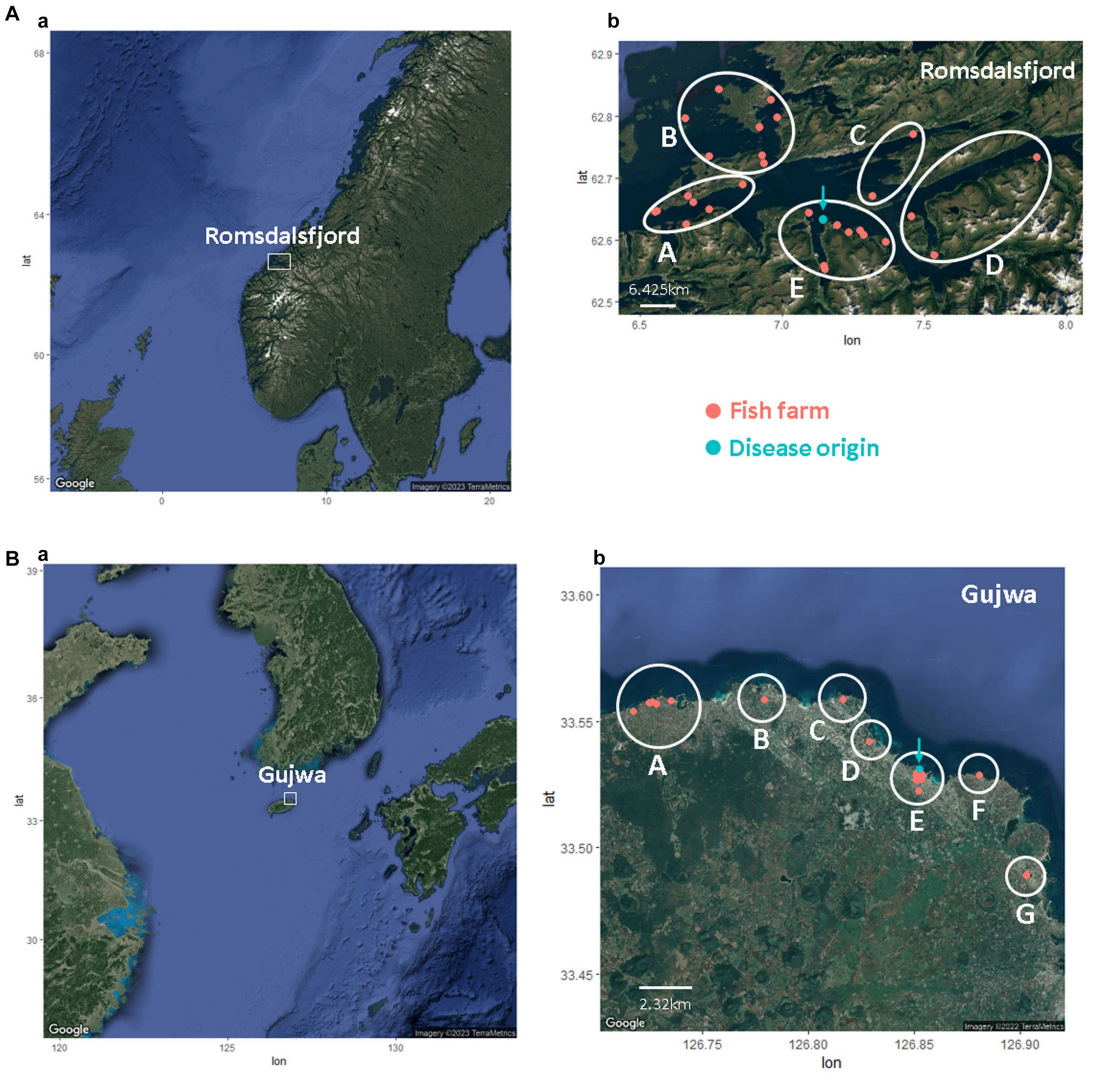
The location of fish farms in Romsdalsfjord **(A)** and Gujwa **(B)**. The white circle indicates the bay management area (BMA) applied in this study. The turquoise dot means the site’s first disease outbreak simulated in this study.

Romsdalsfjord, a semi-closed fjord in Norway, is a famous fish farm area (production zone 5) important for fish production; it includes ~30 fish farms selected for the simulation in this study (25). Likewise, Gujwa, located on Jeju Island, where many olive flounder are cultured, was chosen in this study. Regardless of facilities and systems in fish farms, all simulations in this study assume one single epidemiological pen if they are on the same fish farm. It is also assumed that each fish farm has cultured around 135,000 fish, given the mean value of the average number of flounder annually produced on Jeju Island divided by the total number of fish farms. The geographical data, including the fish farms, fish species, address, and GPS information on Jeju Island, was attained from a previous study (25) and the Korean public data portal (DATA.go.kr),^1^ Geocode, and Google maps (Google, CA, USA). The fish farms in the region of Romsdalsfjord and Gujwa, the sites of the most intensive farming, were used for simulations by different pathogen transmission powers (Figure 1). The bay management area (BMA) was arbitrarily divided for the simulations in this study based on the location of fish farms in Romsdalsfjord and Gujwa. Five (A – E) and seven BMAs (A – G) were allocated to the Romsdalsfjord and Gujwa regions. The beeline distance between fish farms and the site where the disease emerged is calculated using the following formula.


$$Lat1=\frac{\left(1st\;\mathrm{farm}\;\mathrm{latitude}\times \pi \right)}{180}\;Lon1=\frac{\left(1st\;\mathrm{farm}\;\mathrm{longitude}\times \pi \right)}{180}$$

$$Lat2=\frac{\left(2nd\;\mathrm{farm}\;\mathrm{latitude}\times \pi \right)}{180}\;Lon2=\frac{\left(2nd\;\mathrm{farm}\;\mathrm{longitude}\times \pi \right)}{180}$$

$$\mathrm{Beeline}\;\mathrm{distance}\;\mathrm{between}\;1st\;\mathrm{and}\;2nd\;\mathrm{fish}\;\mathrm{farm}\;\left(m\right)$$

(1) $$=6378.137\times \mathrm{Acos}\left(\begin{array}{l} \cos\left(Lat1\right)\times \cos\left(Lat2\right)\\ \times \cos\left(Lon2-Lon1\right)\\ +\sin\left(Lat1\right)\times \sin\left(Lat2\right) \end{array}\right)\times 1000$$

In the case of different fish farms sharing the same GPS information, it was assumed that the distance between farms was around 40 – 400 m. In this study, a total of 460 (_29_C_2_) and 2,145 (_66_C_2_) pairwise beeline distances were obtained for fish farms in Romsdalsfjord (29 fish farms) and Gujwa (66 fish farms), respectively. The transmission of a bacterial pathogen, *M. viscosa*, in Romsdalfjord, Norway, and *S. parauberis* in the Gujwa region, South Korea, among different farms, was simulated with different scenarios of transmission.

### 2.2. First scenario of transmission by different *ScalingInf*

Transmission of *M. viscosa* between different farms in Romsdalfjord, Norway, and *S. parauberis* between different farms in the Gujwa region, South Korea, were simulated with the first scenario of transmission by different *ScalingInf*. All simulations and adjusting parameters were carried out based on a spatiotemporal hybrid simulation model that incorporates both an agent-based and a susceptible-latent-subclinical-clinical-removed model using DTU-DADS-Aqua modified from DTU-DADS-ASF in R version 4.2.1 (21–24, 26). Since the study of how far a bacterial pathogen (*M. viscosa* or *S. parauberis*) can be transmitted through seawater has not been well documented and might be different depending on strains and environments, three different values for *ScalingInf*, a parameter of pathogen transmission with distances between farms (−1, −1.8, and − 2.6; as the high, moderate, and lower transmission power following the distance between fish farms for bacterial disease), were used for each simulation and compared (Simulation A, B, and C) (Supplementary Table S1). In the region of Romsdalsfjord, the disease transmission was additionally simulated using the default value −0.42 *ScalingInf* of condition that can spread to other BMA for the reference (Simulation_Null). This study simulates cases of disease outbreak originating in BMA – E in both Romsdalsfjord, Norway, and the Gujwa region, South Korea, as shown in Figure 1 at the time = 0 (0 days). The value of host susceptibility to bacterial disease (*RelSusceptibility*) and percentage of dead fish (or those that recovered and were no longer infected without shedding pathogens) was set to 1 (100%). For the surveillance and depopulation option, the values for the time between two surveillance visits for herds in a surveillance zone (*ZSurVisit*), the number of surveyed farms every day (*CapSurvay*), and culling capacity per day were set to 30, 5, and 20,000, respectively. All simulations were run for 100 iterations for 365 days. The time of spreading bacterial pathogens was estimated for every iteration and day to predict the possibility of infection in each farm from 1 to 365-day(s) post emerging disease (dpe) throughout 100 iterations.

### 2.3. Second scenario of transmission by *RelSusceptibility*

Although the first scenario assumed a *RelSusceptibility* 1.0 for bacterial infection, the host health conditions, including vaccine and resistant breeds, may increase host resistance against specific pathogens. In order to check the effect of the level of host susceptibility that can affect disease transmission, the disease spread simulations were carried out under different *RelSusceptibility* (1.0, 0.5, 0.2, and 0.05) with the same *ScalingInf* (−1.0) (simulation A, D, E, and F). Except for *RelSusceptibility*, values for all parameters and the origin site of emerging disease were the same as in the first scenario (Supplementary Table S2).

### 2.4. SEIR modeling in a single farm under the application of different host susceptibility

In order to estimate the impact of a disease outbreak on each farm post-disease outbreak, Susceptible-Exposed-Infectious-Recovered/Removed (SEIR) was carried out with varying levels of immunized fish. The disease outbreak was assumed to have started from 1% of the total number of fish exposed to the pathogens in the same fish farm. In detail, the transmission coefficient, latent, recovered period, and daily mortality were assumed to be 0.4, 5 days, 10 days, and 5% for the groups of different numbers of immunized fish (0, 50, 80, and 95%). For fish that were initially immune or had recovered after infection, it was assumed that they would not be infected during the simulation period.

### 2.5. Third scenario of transmission by the different sites of disease outbreak

Depending on the disease transmission control strategy and the location of the fish farm where the disease first breaks out, the prognosis and pattern of disease transmission among all fish farms in the Romsdalsfjord and Gujwa regions can be different. One fish farm in each BMA (A – E) was randomly selected as the first disease outbreak site for each simulation to evaluate the impact of disease transmission by the initial site to start disease transmission. The susceptibility and percentage of fish killed by the disease after infection (*PerDeadAnim*) were assumed as 0.8 (80%) at all simulations for the third scenario, but the same *ScalingInf* was applied as in the second scenario (Supplementary Table S3). For minimizing the influence of disease transmission control, the culling capacity (depopulation) for simulations G, H, I, J, K, L, and M that assumed disease outbreak in Romsdalsfjord (BMA A, B, C, D, E) and Gujwa (BMA A, B, C, D, E, F, and G) was set to none (FALSE), whereas simulations N, O, P, Q, R, S, and T were set to the total number of fish in a single fish farm with the same number of fish (135,000 fish) that can be culled every day if the disease is detected under the following conditions of surveillance control shown in Supplementary Table S3. The possibility of infection day by day post emerging disease from BMA A – G was calculated in each farm under or without control of the severe disease transmission (culling capacity: 135,000 fish day^−1^). Also, the elapsed days of the first infection and detection time in each farm were simulated and compared from 100 iterations in simulation N – T. From the result of post-simulation, the number of infection counts out of 100 iterations in each farm under and without the use of culling strategy were statistically compared based on Chi-square analysis using a ‘gmodels’ package in R studio (V. 4.2.1) (27), and *p*-values of less than 0.05 were considered fish farms significantly affected by the depopulation strategy.

### 2.6. Data analysis and visualization

The location of each farm, the possibility of infection, the first time of infection and detection, and SEIR simulations in an individual farm were calculated and visualized in Google Maps using ‘ggmap’, ‘dplyr’, ‘ggplot2’, ‘devtools’, ‘rvest’, ‘plyr’, and ‘deSolve’ packages and ‘DTU-DADS-Aqua’ scripts in R (version 4.2.1) (21–24, 26, 28–33). The results of disease transmission at each fish farm from the 1st to the 365th day post-exposure in all simulations were visualized in the video file by rendering all figures in chronological order using Microsoft photo (Microsoft Corporation, IL, USA).

## 3. Results

### 3.1. The possibility of disease transmission in the Romsdalsfjord and Gujwa regions by different *ScalingInf* values (scenario 1)

**Figure 2:**
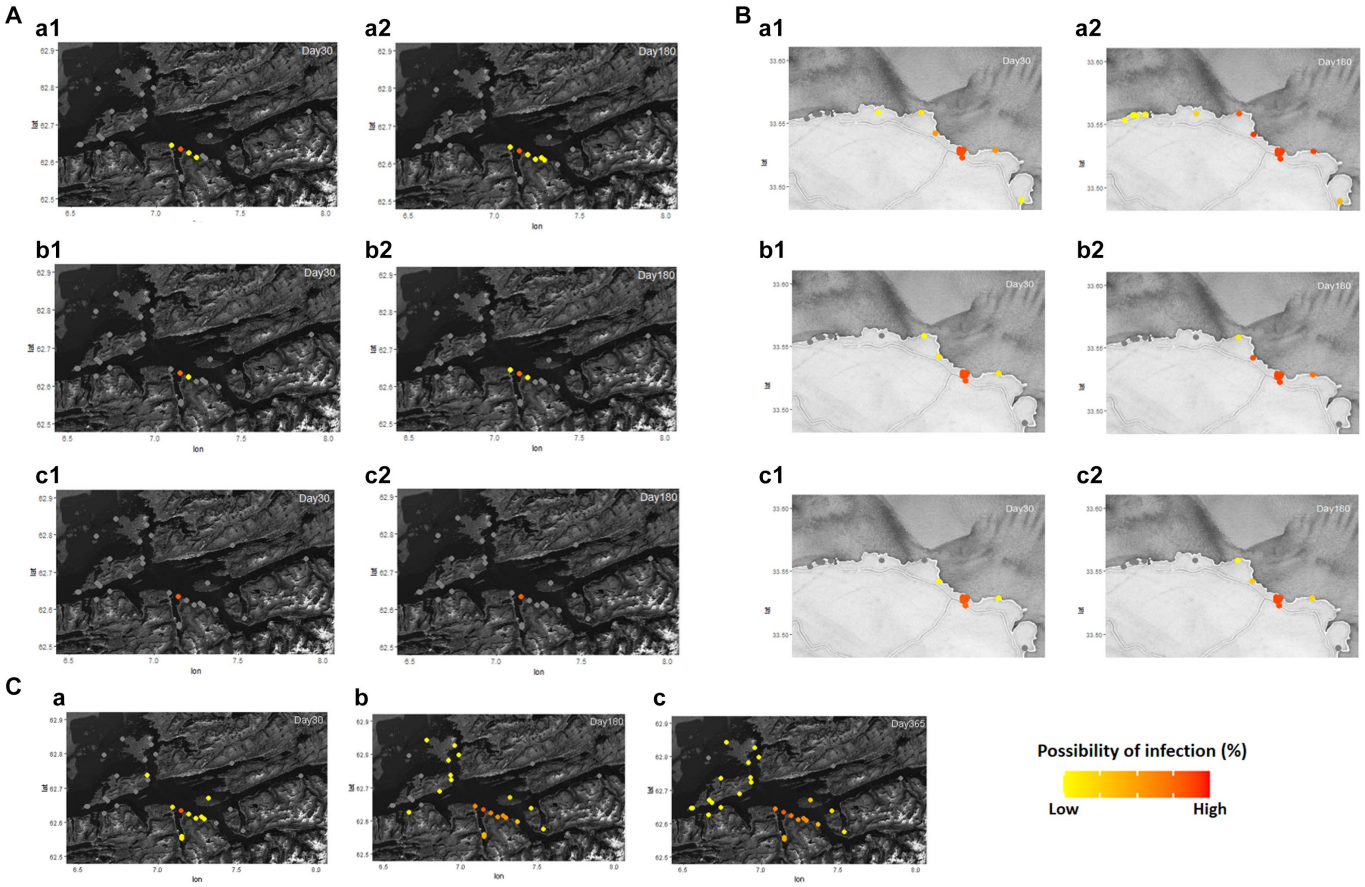
Different distance scaling factors (*ScalingInf*; −1.0, −1.8, and − 2.6) in Romsdalsfjord **(A)** and Gujwa **(B)** were applied to simulate the infectious disease transmission between fish farms by the condition of simulations A, B, and C, respectively (Supplementary Table S1). Regardless of region, **a1** and **a2** show the possibility of infection in each farm simulated at 30 and 180 days post emerging disease (dpe) with −1.0 scaling factor, and **b1** and **b2** show the possibility of infection simulated at 30 and 180 dpe with −1.8 scaling factor. **c1** and **c2** indicate the possibility of infection in each farm at 30 and 180 dpe with −2.6 scaling factor. *ScalingInf* − 0.42 was applied in the Romsdalsfjord region **(C)** by the condition of Simulation_Null, and the possibility of infections in each farm simulated at 30, 180, and 365 dpe are illustrated in **(C-a–c)**. The estimation of the spreading disease pattern from 1–365 dpe is available in Supplementary Video S1.

The possibility of disease infection in each region and farm under the different values of *ScalingInf* was simulated in Supplementary Video S1 and Figure 2. In Romsdalsfjord, the pattern of disease transmission between fish farms is confined to the same BMA, and even the adjacent farms show a little possibility of infection. In particular, in all cases, the disease did not spread to other farms until 180 days post emerging disease (dpe) when −2.6 *ScalingInf* was applied in Romsdalsfjord (Figure 2A). On the other hand, when it comes to the Gujwa region, the value for −1.0 *ScalingInf* can make the disease spread from BMA E to farms in BMA B, C, D, and F even during the early disease transmission phase. When −1.0 *ScalingInf* is applied in the simulation, there is a low probability of disease transmission to even BMA A, which is the furthest site from the initial disease outbreak site until 180 dpe (Figure 2B). With −1.8 and − 2.6 *ScalingInf* values, the pattern of disease transmission is not as dramatic as in the case of −1.0 *ScalingInf*, but still, the disease can be transmittable to the fish farms in adjacent BMA within a month. Simulations employing −1.8 and −2.6 *ScalingInf* do not result in any possibility of disease transmission to farms situated in BMA A, B, and G until 180 dpe. (Figure 2B). Despite the same gap in *ScalingInf* between simulations A and B (Δ0.8) or simulations B and C (Δ0.8), the possibility of infection in each fish farm between simulations B and C is not as large as in simulations A and B. However, the higher *ScalingInf* can spread the disease widely to different BMAs (Figure 2C).

### 3.2. The possibility of disease transmission in the Romsdalsfjord and Gujwa areas by host susceptibility (*RelSusceptibility*)

**Figure 3:**
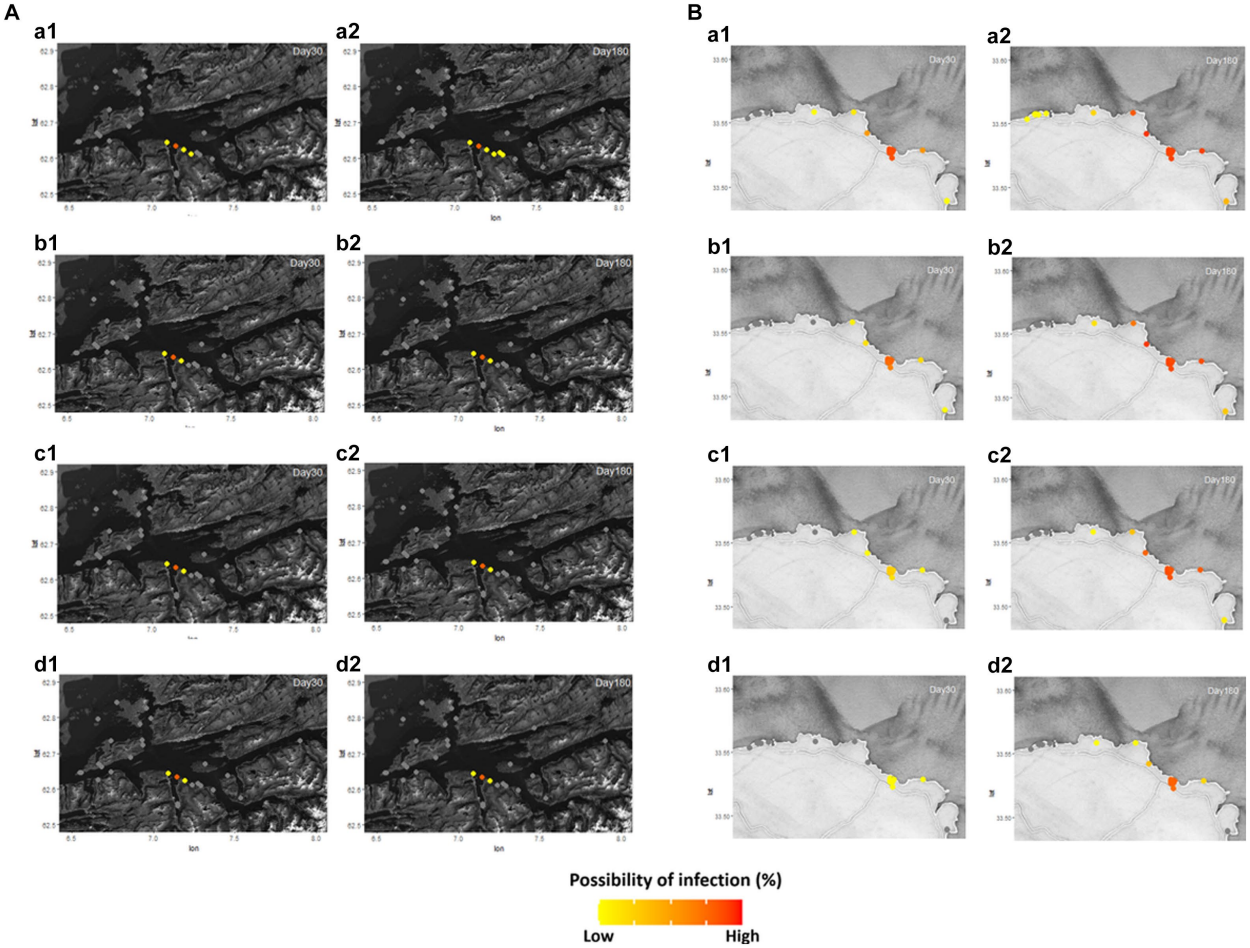
Different disease host susceptibility (*RelSusceptibility*; 1, 0.5, 0.2, and 0.05) was applied in Romsdalsfjord **(A)** and Gujwa **(B)** to simulate the infectious disease transmission following the conditions of simulations A, D, E, and F (Supplementary Table S2). Regardless of region, **a1** and **a2** describe the possibility of infection in each farm simulated at 30 and 180 days post emerging disease with 1.0 *RelSusceptibility*, and **b1** and **b2** show the possibility of infection simulated at 30 and 180 dpe with 0.5 *RelSusceptibility*. **c1** and **c2** indicate the possibility of infection in each farm at 30 and 180 dpe under the condition of 0.2 *RelSusceptiblity*, and **d1** and **d2** describe the possibility of infection in each farm at 30 and 180 dpe under the application of 0.05 *RelSusceptibility*. The estimation of the spreading disease pattern from 1–365 dpe is available in Supplementary Video S2.

The pattern of disease transmission was simulated in the Romsdalsfjord and Gujwa regions based on the host susceptibility, which can vary due to factors such as vaccination and disease history. Since the disease transmission in the Romsdalsfjord region is not remarkable between fish farms, little change is observed by the different *RelSusceptibility* (Figure 3A). On the other hand, in the Gujwa region, where the distance between fish farms is relatively narrow, different *RelSusceptibility* greatly affected disease transmission patterns. When the host had 100% susceptibility (1.0 *RelSusceptibility*) in Gujwa, it showed a high possibility of infection, not only in BMA E but also in BMA D and F regions within 30 dpe. Although the possibility of infection was low, it was predicted that all fish farms in the Gujwa region had a chance of being infected. From a time-wise perspective, no significant difference in the possibility of infection was observed between 180 and 365 dpe under 1.0 *RelSusceptibility* (Figure 3B; Supplementary Video S2B). When it comes to applying lower *RelSusceptibility* (0.5, 0.2, and 0.05), the number of infected fish farms decreased in both the early and late phases of infection and the speed of disease spread was slower than in the high *RelSusceptibility* group (Figure 3B; Supplementary Video S2B). For example, the average time of first infection in the 1.0 *RelSusceptibility* group (simulation A) for all fish farms in BMA E was approximately 17.2 ± 0.6 days. However, it was extended to 22.9 ± 0.7, 36.2 ± 1.3, and 90.1 ± 3.4 days in the 0.5, 0.2, and 0.05 *RelSusceptibility* groups (simulation D, E, and F), respectively. Moreover, no infection was predicted in any fish farms belonging to the BMA A region at 180 dpe, except for the full susceptibility group (simulation A).

**Figure 4:**
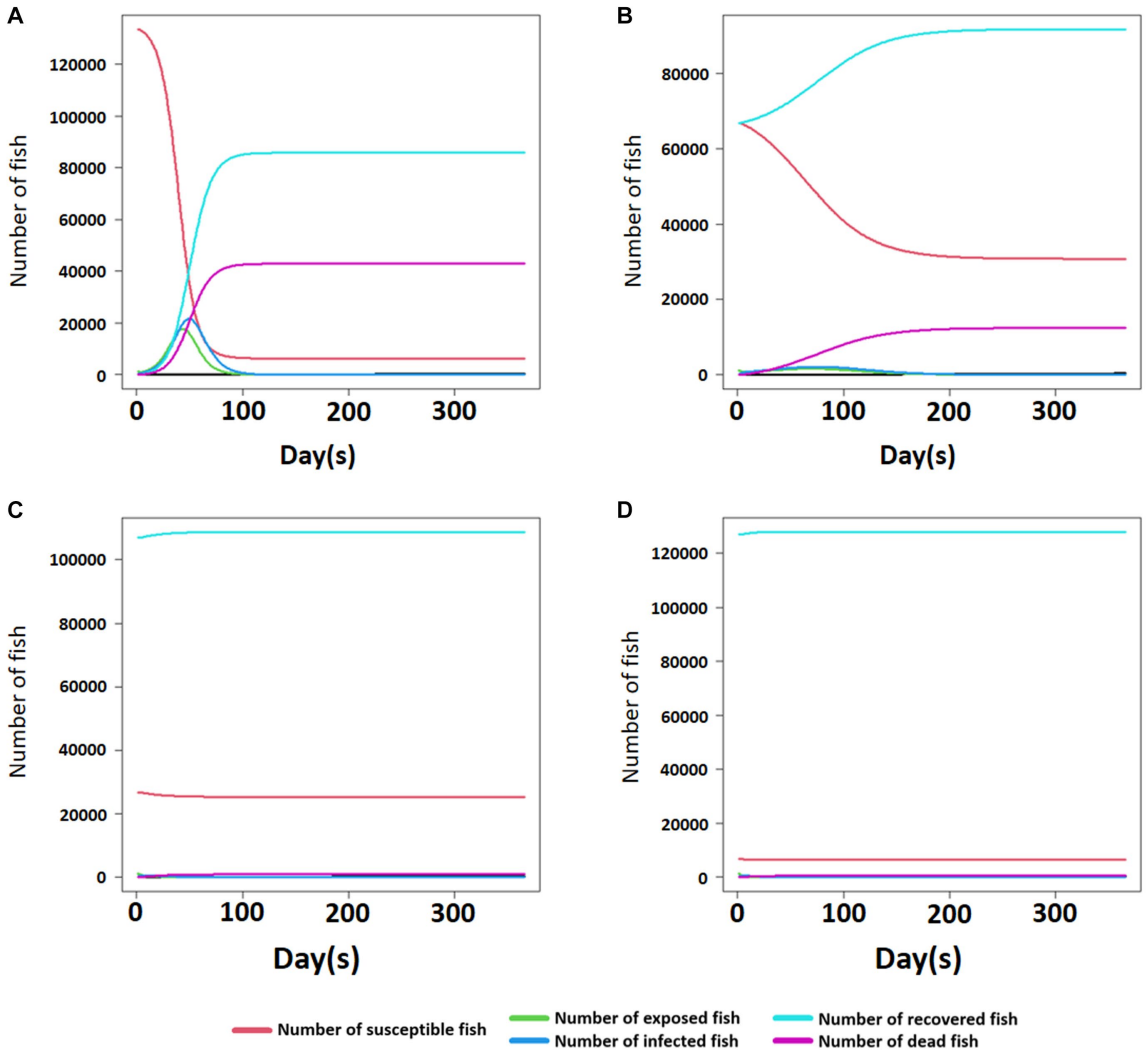
The number of susceptible, exposed, infected, recovered, and dead fish with the time of post-infectious bacterial pathogen exposure in the fish farm where 0, 50, 80, and 95% of immunized fish out of 135,000 fish exist **(A–D)**.

The results of the SEIR simulation for the damage of a single fish farm after a disease outbreak showed that in the absence of immunized fish (susceptibility: 100%), the estimated mortality was around 42,900 fish. However, when 50, 80, and 95% of the fish in the herd had acquired immunity at the beginning, it was predicted that approximately 12,500, 900, and 500 fish would die, respectively (see Figure 4). Likewise, the number and proportion of susceptible and immunized fish considerably changed in the group without immunized fish (susceptibility: 100%) after the disease outbreak (Figure 4A). However, fewer changes were observed following a higher proportion of immunized fish (50, 80, and 95%) at the beginning (Figures 4B–D).

### 3.3. The possibility of disease transmission by the site of the disease outbreak

**Figure 5:**
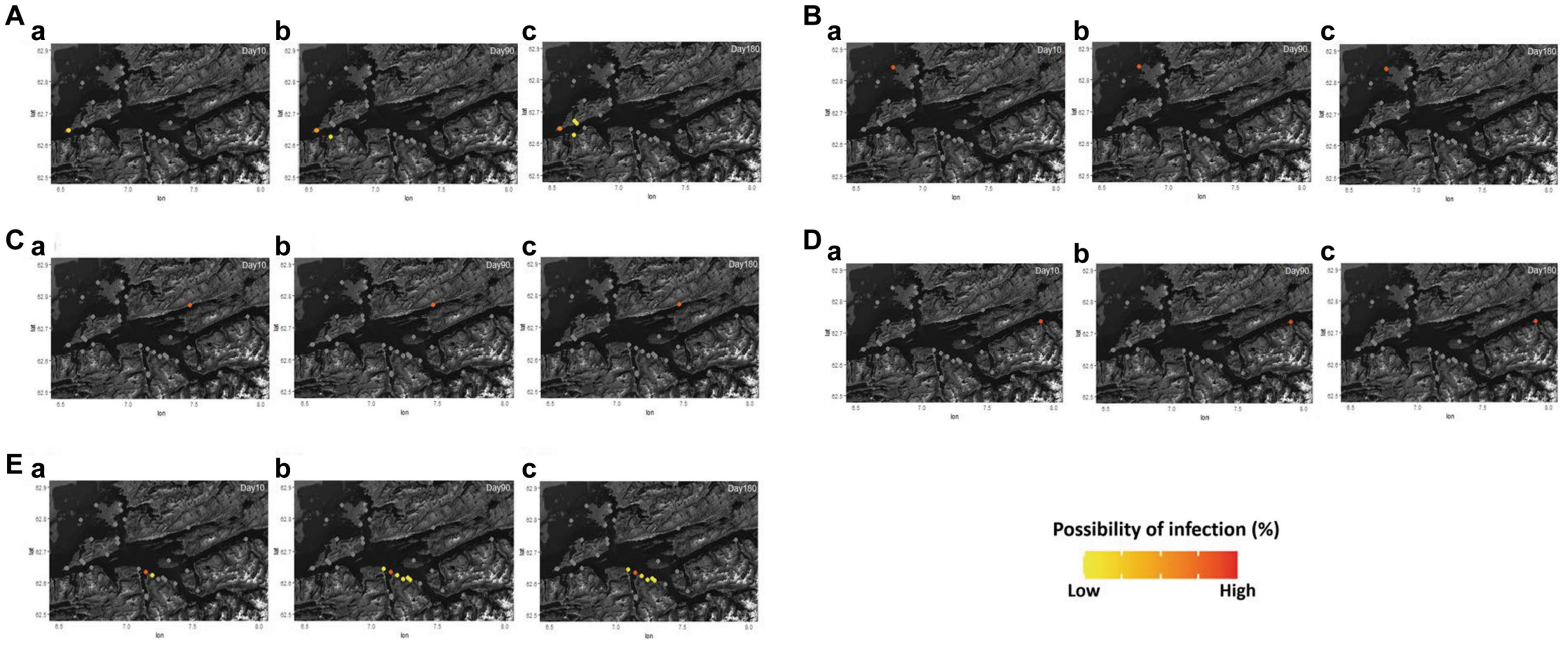
The pattern of bacterial disease transmission between fish farms with the assumption of the first disease outbreak in BMA A, B, C, D, and E under the condition of simulation G, H, I, J, and K in Romsdalsfjord **(A–E)** (Supplementary Table S3). The possibility of infection in each farm is shown at 10 **(A-a,B-a,C-a,D-a,E-a)**, 90 **(A-b,B-b,C-b,D-b,E-b)**, and 180 **(A-c,B-c,C-c,D-c,E-c)** dpe. The estimation of the disease spreading pattern from 1–365 dpe is available in Supplementary Video S3.

The above two scenarios have assumed that the disease originated from the seashore or fish farm on BMA E. However, the third scenario was designed for the situation when the disease occurred at one of the fish farms in each BMA and simulated the pattern of disease transmission between fish farms. In Romsdalsfjord, except for BMA A and E, the disease with the transmission power simulated in this study did not significantly influence adjacent fish farms within a BMA (Figure 5; Supplementary Video S3).

**Figure 6:**
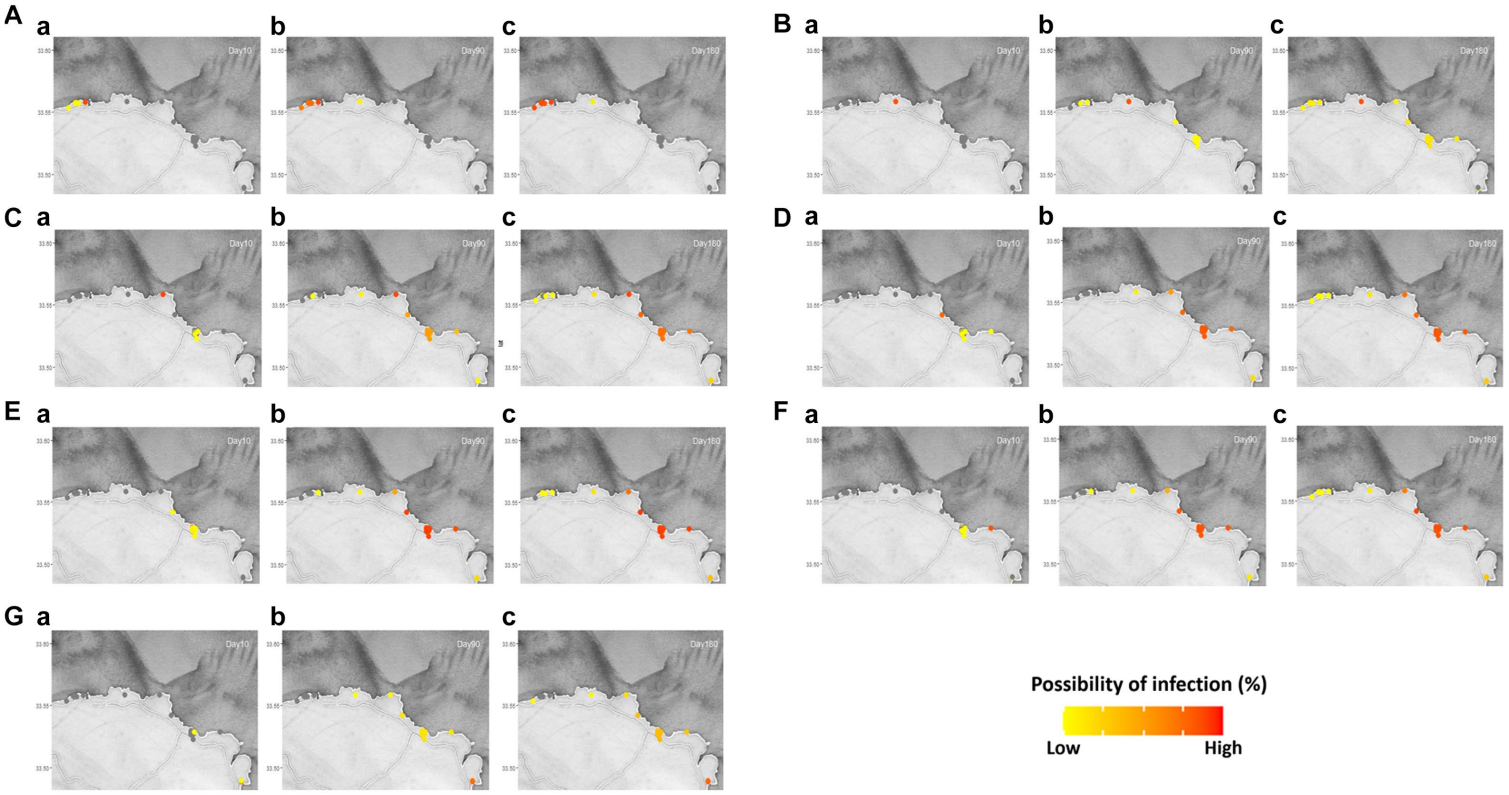
The pattern of bacterial disease transmission with the assumption of first disease outbreak in BMA A, B, C, D, E, F, and G in the Gujwa region under the condition of stimulation G, H, I, J, K, L, and T **(A–G)**. The possibility of infection in each farm is shown at 10 **(A-a,B-a,C-a,D-a,E-a,F-a,G-a)**, 90 **(A-b,B-b,C-b,D-b,E-b,F-b,G-b)**, and 180 **(A-c,B-c,C-c,D-c,E-c,F-c,G-c)** dpe. The estimation of the spreading disease pattern from 1–365 dpe is available in Supplementary Video S4.

The results of simulations that assume the initial disease outbreaks in BMA C, D, E, or F showed the disease could spread to the fish farms located in BMA E, which has the highest number of fish farms in the Gujwa region. Furthermore, the subsequent patterns of disease transmission were similar across these simulations (Figure 6; Supplementary Video S4). When it comes to simulation M, since the origin of the disease is in BMA G, relatively far from BMA E, the time of infection and the possibility of infection at fish farms in BMA E is relatively slower and lower than in simulations I, J, K, and L (Figure 6; Supplementary Video S4). In addition, there was a pattern of faster and earlier disease transmission to BMA E’s fish farms than other adjacent BMA(s), which is a shorter distance from the site where the disease first appeared in simulations H, D, and M (Figure 6; Supplementary Video S4).

### 3.4. Comparison of the possibility of infection in each fish farm following disease transmission control strategy

**Figure 7:**
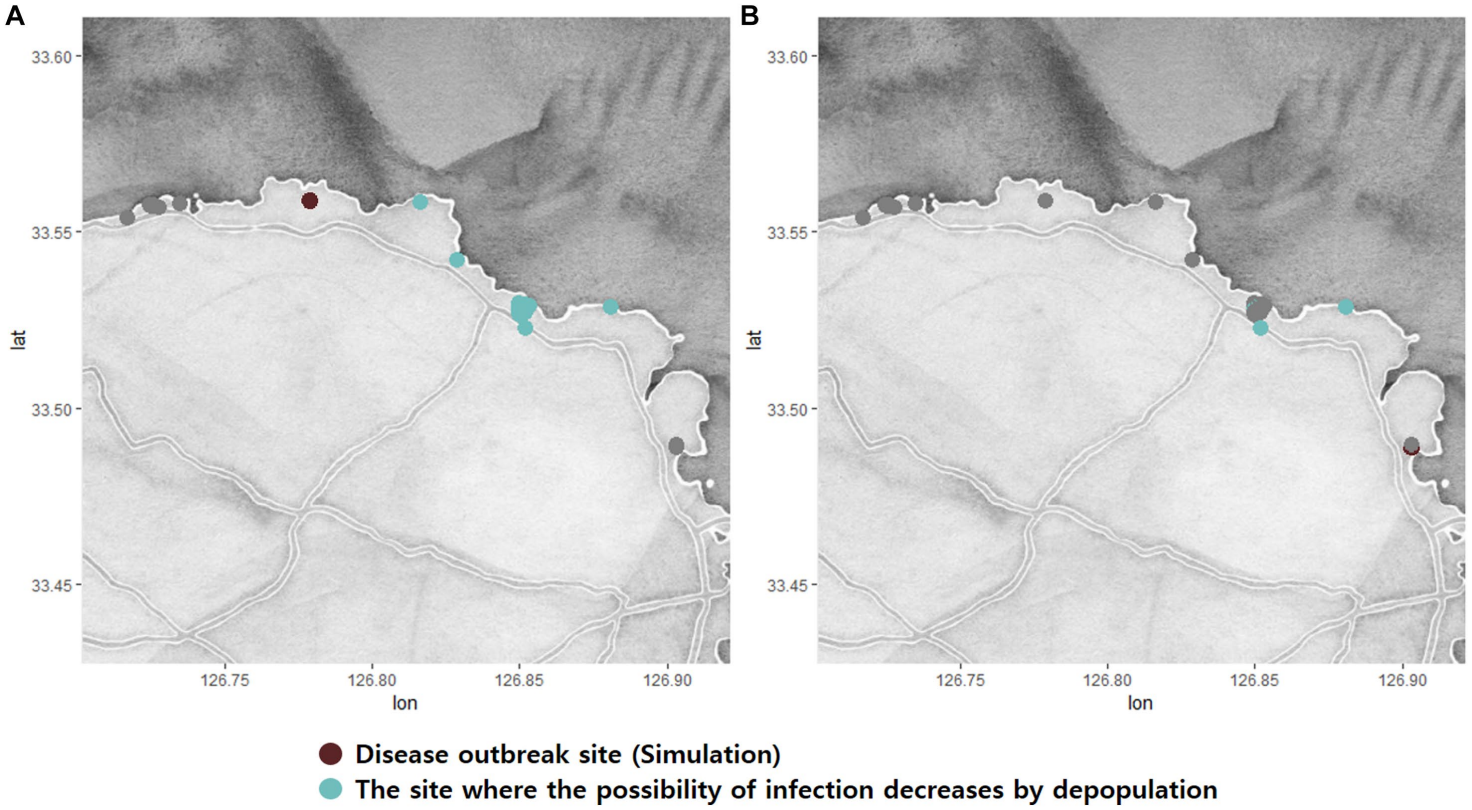
Simulations O **(A)** and T **(B)** show the possibility of significantly reducing the rate of infection through depopulation control in the Gujwa region. The green dots indicate the fish farm where the possibility of infection decreases significantly by Chi-square analysis (*p* < 0.05).

**Figure 8:**
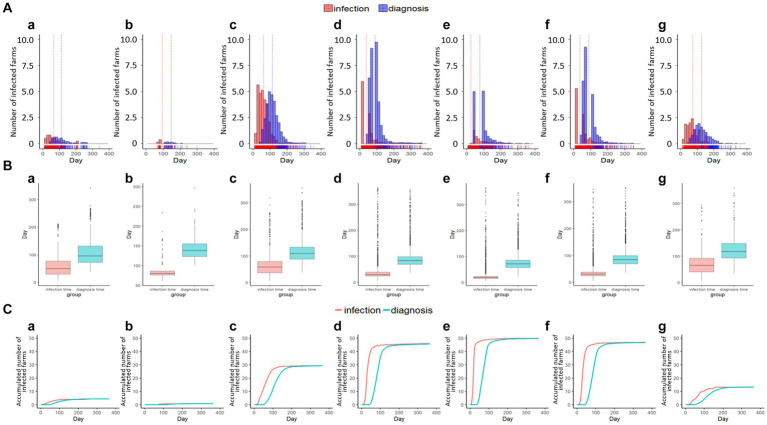
The number of infected and detected farms with the time of emerging disease based on simulations N, O, P, Q, R, S, and T shown in the histogram with vertical lines for the mean value of infection and diagnosis time **(A-a–g)**, box plot **(B-a–g)**, and cumulative curve **(C-a–g)** in the Gujwa region.

The simulations with and without culling the fish were compared with the assumption that the first disease outbreak was in the same BMA to evaluate the effect of depopulation as preemptive quarantine measures. Since the infectious disease is not extensively spreading in Romsdalsfjord, it is difficult to evaluate the effect of depopulation (Table 1). However, more differences were observed in the Gujwa region. The effects and outcomes of the depopulation strategy, which utilized 135,000 for a daily culling capacity as a quarantine measure, varied significantly depending on the location where the disease initially emerged. In the cases of initial disease outbreaks at BMA A, C, D, E, and F, the depopulation strategy had very little impact on the possibility of infections among fish farms in the Gujwa region (Table 2). On the other hand, the simulation where we set the first disease outbreak farm at BMA B and G showed the depopulation strategy could lead to a significant decrease in the possibility of infection in fish farms (Figure 7; Table 2). In particular, when the disease first occurred in the BMA B, it was predicted that the depopulation strategy contributed to significantly reducing the possibility of infection to 0–1% in more than 85% of fish farms in the Gujwa region (Table 2). The strategy of culling fish (135,000 fish day-1) can help to reduce the total number of infected fish farms and diagnosed farms when the pathogen originated from BMA B and G, which implies that the effect of culling the fish can vary depending on the origin site of a disease outbreak (Figure 8). In addition, the expected number of infected fish farms and the time (day) of transitioning to the plateau stage for the curve for cumulative infected and diagnosed fish farms were different according to the site of the first disease outbreak.

**Table 1:** The possibility of infection for the fish farm(s) belonging to each BMA (A–E) with and without depopulation (mean ± standard deviation) under the different sites of disease outbreak in the Romsdalsfjord region.

Disease outbreak	BMAID	Without Cull (FALSE; %)	Cull (135,000; %)	Chisq*	Disease outbreak	BMAID	Without Cull (FALSE; %)	Cull (135,000; %)	Chisq*
**A**	A	20.3 ± 33	17.9 ± 30	(0/7)	**B**	A	0 ± 0	0 ± 0	(0/7)
	B	0 ± 0	0 ± 0	(0/8)		B	10.4 ± 29.3	10.4 ± 29.3	(0/8)
	C	0 ± 0	0 ± 0	(0/2)		**C**	0 ± 0	0 ± 0	(0/2)
	D	0 ± 0	0 ± 0	(0/3)		**D**	0 ± 0	0 ± 0	(0/3)
	E	0 ± 0	0 ± 0	(0/9)		**E**	0 ± 0	0 ± 0	(0/9)
**C**	A	0 ± 0	0 ± 0	(0/7)	**D**	A	0 ± 0	0 ± 0	(0/7)
	B	0 ± 0	0 ± 0	(0/8)		B	0 ± 0	0 ± 0	(0/8)
	C	38.5 ± 54.4	38.5 ± 54.4	(0/2)		C	0 ± 0	0 ± 0	(0/2)
	D	0 ± 0	0 ± 0	(0/3)		D	29 ± 50.2	29 ± 50.2	(0/3)
	E	0 ± 0	0 ± 0	(0/9)		E	0 ± 0	0 ± 0	(0/9)
**E**	A	0 ± 0	0 ± 0	(0/7)					
	B	0 ± 0	0 ± 0	(0/8)					
	C	0 ± 0	0 ± 0	(0/2)					
	D	0 ± 0	0 ± 0	(0/3)					
	**E**	**15 ± 25.9**	**10.8 ± 26.5**	**(1/9)**					

All models are simulated based on a year (365 days). The percentage (%) in the table indicates the count of iterations, out of 100 simulation runs, in which infections occurred in fish farms belonging to each BMA region within 365 dpe. Asterisk (*) indicates the number of fish farms that significantly change the possibility of infection out of the total number of fish farms in each BMA based on a Chi-square analysis (*p* < 0.05). Bold letters indicate BMAs where a significant decrease in the possibility of infection, affecting at least one fish farm, is observed.

**Table 2:** The possibility of infection for the fish farm(s) belonging to each BMA (A–G) with and without depopulation (mean ± standard deviation) under the different sites of disease outbreak in the Gujwa region.

Disease outbreak (BMA)	BMAID	Without Culling (FALSE; %)	Cull (135,000; %)	Chisq*	Disease outbreak (BMA)	BMAID	Without Culling (FALSE; %)	Cull (135,000; %)	Chisq*
**A**	A	86.2 ± 1.9	79 ± 5	(0/6)	**B**	A	11.5 ± 0.8	3.0 ± 0.0	(0/6)
	B	10.0	13.0	(0/1)		B	84.0	84.0	(0/1)
	C	1.0	1.0	(0/1)		**C**	**13.0**	**1.0**	**(1/1)**
	D	0.0	1.0	(0/1)		**D**	**16.0**	**1.0**	**(1/1)**
	E	0.0	0.6 ± 0.5	(0/53)		**E**	**16.8 ± 0.4**	**1.0 ± 0.0**	**(53/53)**
	F	0.0	0.0	(0/1)		**F**	**14.0**	**0.0**	**(1/1)**
	G	0.0	0.0	(0/2)		G	3.5 ± 0.7	0.0	(0/2)
**C**	A	2.7 ± 0.5	5.7 ± 0.5	(0/6)	**D**	A	2.0 ± 0.0	6.5 ± 0.5	(0/6)
	B	24.0	16.0	(0/1)		B	17.0	26.0	(0/1)
	C	88.0	88.0	(0/1)		C	75.0	74.0	(0/1)
	D	83.0	55.0	(0/1)		D	76.0	76.0	(0/1)
	E	79 ± 2.5	51.5 ± 2.1	(0/53)		E	81.6 ± 2.4	81.1 ± 2.4	(0/53)
	F	79.0	52.0	(0/1)		F	81.0	80.0	(0/1)
	G	37.0 ± 0	18.5 ± 0.7	(0/2)		G	38.0 ± 1.4	35.5 ± 2.1	(0/2)
**E**	A	2.8 ± 1.2	2.8 ± 0.8	(0/6)	**F**	A	2.7 ± 0.5	2.2 ± 0.4	(0/6)
	B	21.0	16.0	(0/1)		B	19.0	15.0	(0/1)
	C	77.0	74.0	(0/1)		C	72.0	72.0	(0/1)
	D	91.0	90.0	(0/1)		D	88.0	86.0	(0/1)
	E	88.5 ± 3.0	88.5 ± 2.9	(0/53)		E	84.6 ± 2.7	83.2 ± 2.5	(0/53)
	F	90.0	90.0	(0/1)		F	84.0	84.0	(0/1)
	G	40.5 ± 0.7	47.5 ± 0.7	(0/2)		G	41.5 ± 2.1	40.0 ± 0.0	(0/2)
**G**	A	3.5 ± 0.5	1.0 ± 0.0	(0/6)					
	B	12.0	5.0	(0/1)					
	C	38.0	19.0	(0/1)					
	D	45.0	23.0	(0/1)					
	**E**	**42.8 ± 1.7**	**22.4 ± 0.7**	**(5/53)**					
	**F**	**43.0**	**20.0**	**(1/1)**					
	G	84.0 ± 5.7	77.5 ± 13.4	(0/2)					

All models are simulated based on a year (365 days). The percentage (%) in the table indicates the count of iterations, out of 100 simulation runs, in which infections occurred in fish farms belonging to each BMA region within 365 dpe. Asterisk (*) indicates the number of fish farms that significantly change the possibility of infection out of the total number of fish farms in each BMA based on a Chi-square analysis (*p* < 0.05). Bold letters indicate BMAs where a significant decrease in the possibility of infection, affecting at least one fish farm, is observed.

## 4. Discussion

The modeling and simulations used in this study cannot reflect the real situation with 100% accuracy due to insufficient data and/or the assumption of many epidemiological factors, such as the number of fish and fish farms, host stages, and disease history that affect disease susceptibility, various environment conditions (e.g., tide, oceanic currents), etc. Nevertheless, evaluating and predicting the infection risk in a timely manner, taking into account geographical characteristics, is crucial for sustainable aquaculture as it minimizes risks to the ocean environment and biological diversity (23–25). Accordantly, simulating the best and worst-case scenarios while considering the multi-dimensional factors that can significantly impact the spread of disease can be immensely helpful in preparing and efficiently counteracting future disease outbreaks. In this study, we aimed to predict the potential impact and extent of bacterial disease transmission in the Romsdalsfjord and Gujwa regions. To achieve this goal, we considered several crucial factors that affect the spread of diseases, including *ScalingInf*, *RelSusceptibility*, the distance between farms, and culling capacity. Specifically, we focused on evaluating the epidemiological impact of the distance between fish farms in regions where fish are intensively cultured but have different mean distances between farms. Given that the mean distances between fish farms are ~24 and ~ 8 km in the Romsdalsfjord and Gujwa regions, excluding the fish farm reported in the same GPS site, a comparison of both disease transmission patterns could provide the epidemiological influences of the distance between fish farms on disease transmission. Based on the simulations, the distance between fish farms in Romsdalsfjord is ~3 times longer than Gujwa, and the results confirmed that the distance between farms could have a significant effect in preventing disease against waterborne mediated transmission. However, it does not mean that distantly located farms are protected against the disease because the disease could be spread by pathogens with a higher value of *ScalingInf*, like Simulation_Null, anthropogenic activity, and wild aquatic animals (34–36). In addition, the different geographic environments, semi-closed fjord environments, and open seas could result in epidemiologically different results between regions. Nevertheless, it is certain that the distance between fish farms is the most featured epidemiological factor relevant to disease transmission. In the following discussion, we focus on the impact of other epidemiological factors on disease transmission in the Gujwa region, in addition to the distance between fish farms.

Most pathogens in aquaculture can be delivered and horizontally transmitted by the water. The issue of how long bacterial pathogens can survive and maintain infectivity in the seawater environment should be considered. *ScalingInf* is one of the most influential factors in pathogen dispersion, which is determined by considering various conjugated factors such as water temperature, sun or UV light exposures during the transmission, growth of natural biota in seawater, hydrodynamic variables (tide, water currents) (23, 24, 37, 38). A lower value of *ScalingInf* suggested lower disease transmission between fish farms, and former studies used −0.364 and − 0.6 *ScalingInf* for the simulations of IHNV and ISAV, known as highly infectious viruses (23, 34). However, many important factors are different for the simulation of disease transmission. The biological differences between bacteria and viruses, farming systems, and geographical characteristics can affect disease transmission. Although transmission power can be different depending on species, the transmission of viral diseases is more difficult to control than bacterial diseases due to insufficient vaccines and anti-viral agents (39). In addition, most olive flounder on Jeju Island have been cultured in the land-based aquaculture system that normally filters and disinfects incoming seawater, unlike the sea-cage system, which can also greatly contribute to decreasing pathogen transmission. With the full consideration of these differences, *ScalingInf* was simulated from −1.0 to −2.6 for the Gujwa region and −0.42 to −2.6 for Romsdalsfjord, Norway (23, 24). Although most fish farms in the Gujwa region could be susceptible under the simulation with −1.0 *ScalingInf* from seawater at BMA E, the possibility of disease infection in BMA A, B, and G was relatively lower within 180 dpe. However, Currás et al. (11) successfully showed that ~10^5^ CFU mL^−1^ of *S. parauberis* cannot survive in the seawater environment at 6 and 22°C for longer than 36 days through the direct plate count method, but it can survive longer than 180 days in the marine sediment environment. Furthermore, *S. parauberis* can survive even in seawater for a long time in the form of viable but nonculturable (VBNC) stages (11). Indeed, a high number of copies of *S. parauberis* was detected in seawater off the coast of Gujwa, Jeju Island (4 × 10^7^ copies 100 mL^−1^), but its viable counts were only 4 CFU 100 mL^−1^ (10). This suggests that the bacterial endemic can be much longer than simulations. Moreover, bacterial virulence is different depending on strains and genetic information; even the same strain of bacterial pathogen can be changed when exposed to an environment that can affect multiple phenotypic characteristics (40, 41). The fundamental study to determine *ScalingInf* with the consideration of pathogenicity and other characteristics related to the transmission can help to formulate a more accurate simulation case by case.

Host susceptibility is also one of the important factors for the prevalence of the disease. Several commercial vaccines for the bacterial disease are available, and they have been shown to have a relative survival rate of more or less 50% (5). When simulated with a *RelSusceptibility* 0.5 (50%), the speed of disease transmission between fish farms was slightly slower than the simulation with *RelSusceptibility* 1.0 (100%). *RelSusceptibility* 0.5 did not contribute to reducing disease transmission. Nevertheless, given that the predicted number of dead fish by *S. parauberis* in the 0.5 *RelSusceptibility* group showed more than 3 times lower mortality than 1.0 based on SEIR simulation, it is expected to be of great help in reducing mortality and loss. The simulations with *RelSusceptibility* 0.2 and 0.05 can greatly contribute to both reducing areas where disease can spread and mortality compared to the 1.0 group. However, Hwang et al. (5) conducted a survey and found that approximately 80% of olive flounder in Korean fish farms were vaccinated. Considering the average RPS or protection rate of commercial vaccines (~50%) and the proportion of vaccinated flounder in fish farms (~80%), it has been speculated that the real-world situation could be worse than the simulation assuming a 0.5 *RelSusceptibility*. Currently, most commercial vaccines are based on a formalin-killed vaccine, which could have relatively lower efficacy than other types of vaccines, such as live-vaccine and DNA vaccines (42, 43). The use and development of a more effective vaccine or the development of more robust disease-resistant fish in the future could be an important factor that could significantly reduce mortality and disease transmission. Furthermore, the level of fish stress, nutrient availability, host number, and farm system and maintenance significantly affect the host health condition directly connected to the disease outbreak. Accordantly, the level of *RelSusceptibility* requires overall consideration of these factors by scientists in the relevant fields or fish health biologists.

The pattern of disease spread is also changeable by geographical reasons, such as the location and complexity of fish farms. In general, the disease has been transmitted to adjacent fish farms in most cases at the beginning, and it is very natural, as *S. parauberis* might continuously spread to the surrounding water over time. However, some simulations showed faster disease transmission to fish farms in BMA E, where many fish farms are concentrated but far from the disease outbreak site rather than the fish farms closer to a site where the disease first emerges. For example, even though the disease emerges in simulations H, I, and T originated from BMAs B, C, and G, respectively, a higher likelihood of disease transmission was predicted in BMA E compared to BMAs C, D, or E, despite being geographically closer to the origin of the disease, exhibited a higher potential for transmission due to factors such as the number of fish farms in the BMA and its complexity. The distance between fish farms was also identified as an important contributing factor. Mathematically, it is evident that the possibility of infection in each epidemiological aquaculture unit in BMA E is lower than in the fish farm closer to the disease origin because of the distance. When the lower possibility of infection value was applied to a significantly larger number of fish farms, the presence of many fish farms in BMA E greatly increased the likelihood of a disease outbreak. When the first disease infection occurred, despite a lower probability, faster than other adjacent BMAs, the disease transmission in BMA E might have spread so fast that it was hard to control. Moreover, as a consequence of infection, the BMA E region was predicted to have a much higher number of dead and infected fish than others. These results highlight the importance of spacing out the fish farms and the requirement of coordinated management practices (synchronized stocking, fallowing, etc.) for fish farming. Former studies (44, 45) have emphasized that bacterial pathogens such as *Flavobacterium columnare* infect freshwater fish and can colonize dead fish and spread the pathogens to living fish, which is a more efficient mode of transmission than transmission from diseased to susceptible fish. The simulation applied in this study assumes all dead fish were removed or lost the pathogen infectivity from dead fish from 3 days post-death, which means that the period for which dead fish maintained their infectivity was 2 days (*DayDead*). However, if high mortality within a short period occurs, the situation will be worse than the simulation presented.

Depopulation by culling the fish that are exposed to infectious diseases is one of the strongest strategies for controlling disease transmission. Assessing the potential impact of depopulation can assist in identifying optimal countermeasures in the event of a worsening situation or an outbreak of emerging diseases. It is worth noting that employing the culling strategy does not necessarily yield the same expected effect, and its outcome can vary depending on the location of the disease outbreak. Regrettably, in the Gujwa region, the use of the depopulation strategy did not significantly affect the transmission of the disease from BMA A, C, D, E, and F, as the possibility of infection remained unchanged. The failure to achieve the benefits of depopulation may be primarily due to the gap between the initial infection and the time of detection, despite the influence of various factors such as geographical characteristics, fish population, and surveillance options. Since culling is performed only after diagnosis, the pathogen may have already spread extensively throughout the sea by the time the surveillance system identifies the disease. However, when the disease occurred in the BMA B region at the beginning, which saw the highest effects of depopulation, it was simulated that there was little possibility of transmission to the nearest adjacent farms for 60 dpe. Given the average time gap between the first infection and diagnosis time is around 50 days, adopting a depopulation strategy could potentially eliminate the source of the disease before it spreads to other fish farms. In summary, the effectiveness of depopulation depends significantly on the extent to which the disease has already spread. As Romero et al. (23, 24) simulated previously, the increase in surveillance and control capacity can lead to detecting the disease promptly in each farm. Early detection does not always link to successful depopulation to control the disease, but it can increase the possibility of success.

In conclusion, this study simulated disease transmission under the assumption of varying bacterial disease transmission power in two different regions, Romsdalsfjord in Norway and Gujwa in South Korea. The results revealed that the distance between fish farms is a crucial factor in controlling disease transmission. Specifically, disease transmission in Romsdalsfjord could be controlled within a few fish farms because of the longer distance between fish farms, while the opposite was observed in Gujwa. Depending on the set *ScalingInf* value, the simulation resulted in the spread of disease in either a few fish farms or all fish farms in the Gujwa region. Hence, it is necessary to have a more accurate *ScalingInf* value specific to the infectious agent. The study also highlights the importance of *RelSusceptibility* in disease transmission. The efficacy of the commercially used vaccine (RPS: ~50%) might not significantly affect the overall disease spreading pattern in the Gujwa area, but it can reduce the mortality in each farm by more than three times. Future vaccines with higher efficacy can prevent both disease transmissions between farms and mortality in each farm. All the results presented in this study support that efforts to enhance the host’s disease resistance, such as vaccination, the use of specific disease-resistant breeds, probiotics, etc., not only reduce the damage caused by diseases in individual farms but also significantly contribute to preventing disease transmission among fish farms epidemiologically. In addition, the simulations with the assumption of a different BMA site as the first disease occurred suggest that the depopulation strategy can be highly dependent on geographical characteristics and the control capacity of the surveillance system. While the simulation based on DTU-DADS-Aqua and SEIR might be simplistic, considering various factors affecting the event in the real world, it can still contribute significantly to predicting disease transmission while considering geographical characteristics and the impact of vaccination and surveillance systems in Aquaculture.

## Data availability statement

The original contributions presented in the study are included in the article/Supplementary material, further inquiries can be directed to the corresponding author.

## Author contributions

HR: conceptualization, data curation, methodology, visualization, results analysis, writing, revising, and approving. DK: writing, revising, review, and editing. All authors contributed to the article and approved the submitted version.

## Conflict of interest

The authors declare that the research was conducted in the absence of any commercial or financial relationships that could be construed as a potential conflict of interest.

## Publisher’s note

All claims expressed in this article are solely those of the authors and do not necessarily represent those of their affiliated organizations, or those of the publisher, the editors and the reviewers. Any product that may be evaluated in this article, or claim that may be made by its manufacturer, is not guaranteed or endorsed by the publisher.

## Footnotes

^1^https://www.data.go.kr/data/15056439/fileData.do; as of 2020.02.24.
