# Supplementary material for: Assessments of epidemic spread in aquaculture: comparing different scenarios of infectious bacteria incursion through spatiotemporal hybrid modeling
Source: Front Vet Sci. 2023 Sep 13;10:1205506. doi: 10.3389/fvets.2023.1205506 (PMC10527373; doi:10.3389/fvets.2023.1205506)
Supplement: Supplementary file 1 [file Table_1.docx]

Supplementary Tables

Assessments of Epidemic Spread in Aquaculture: Comparing Different Scenarios of Infectious Bacteria Incursion through Spatiotemporal Hybrid Modeling

HyeongJin Roh^1^*, Dhamotharan Kannimuthu^1^

*** Correspondence:** HyeongJin Roh: [HyeongJin.Roh@hi.no](mailto:HyeongJin.Roh@hi.no)

**Table S1.** Parameter values selected for simulation A, B, and C belonging to the first case of *M. viscosa/* *S. parauberis* outbreak scenario. All simulations are based on a year (365 days) with a different distance scaling factor (*ScalingInf*). Except for the values mentioned in this study, the remaining elements were set to their default value.

|  | **Simulation A** | **Simulation B** | **Simulation C** | **Simulation_Null** |
| --- | --- | --- | --- | --- |
| Iteration | 100 | 100 | 100 | 100 |
| ***ScalingInf*** | **-1.0** | **-1.8** | **-2.6** | **-0.42** |
| Susceptible | 1 | 1 | 1 | 1 |
| PerDeadAnim | 1 | 1 | 1 | 1 |
| Cull capacity | 20,000 | 20,000 | 20,000 | 20,000 |
| Farm size | 135,000 | 135,000 | 135,000 | 135,000 |
| ZSurVisit | 30 | 30 | 30 | 30 |
| Capsurvey | 5 | 5 | 5 | 5 |
| Disease origin (BMA) | E* | E* | E* | E* |
| **Simulation regions** | **Romsdalsfjord & Gujwa** | **Romsdalsfjord & Gujwa** | **Romsdalsfjord & Gujwa** | **Romsdalsfjord** |

Asterisk (*) indicates the origin of *M. viscosa/* *S. parauberis* as shown in Figure 1.

**Table S2.** Parameter values selected for simulation A, D, E, and F belonging case study-2 comparison. All simulations are based on a year (365 days) with a different pathogen susceptible value. Except for the values mentioned in this study, the remaining elements were set to their default value.

|  | **Simulation A** | **Simulation D** | **Simulation E** | **Simulation F** |
| --- | --- | --- | --- | --- |
| Iteration | 100 | 100 | 100 | 100 |
| ScalingInf | -1.0 | -1.0 | -1.0 | -1.0 |
| **Susceptible** | **1** | **0.5** | **0.2** | **0.05** |
| PerDeadAnim | 1 | 1 | 1 | 1 |
| Cull capacity | 20,000 | 20,000 | 20,000 | 20,000 |
| Farm size | 135,000 | 135,000 | 135,000 | 135,000 |
| ZSurVisit | 30 | 30 | 30 | 30 |
| Capsurvey | 5 | 5 | 5 | 5 |
| Disease origin (BMA) | E* | E* | E* | E* |

Asterisk (*) indicates the origin of *M. viscosa*/ *S. parauberis* as shown in Figure 1.

|  | **Simulation G/N** | **Simulation H/O** | **Simulation**  **I/P** | **Simulation J/Q** | **Simulation K/R** | **Simulation L/S** | **Simulation M/T** |
| --- | --- | --- | --- | --- | --- | --- | --- |
| Iteration | 100 | 100 | 100 | 100 | 100 | 100 | 100 |
| ScalingInf | -1.0 | -1.0 | -1.0 | -1.0 | -1.0 | -1.0 | (-)1.0 |
| Susceptible | 0.8 | 0.8 | 0.8 | 0.8 | 0.8 | 0.8 | 0.8 |
| PerDeadAnim | 0.8 | 0.8 | 0.8 | 0.8 | 0.8 | 0.8 | 0.8 |
| **Cull capacity** | **FALSE/135000** | **FALSE/135000** | **FALSE/135000** | **FALSE/135000** | **FALSE/135000** | **FALSE/135000** | **FALSE/135000** |
| Farm size | 135,000 | 135,000 | 135,000 | 135,000 | 135,000 | 135,000 | 135,000 |
| ZSurVisit | 30 | 30 | 30 | 30 | 30 | 30 | 30 |
| Capsurvey | 5 | 5 | 5 | 5 | 5 | 5 | 5 |
| **Disease origin**  **(Romsdalsfjord)** | **A** | **B** | **C** | **D** | **E** | - | - |
| **Disease origin**  **(Gujwa)** | **A** | **B** | **C** | **D** | **E** | **F** | **G** |

**Table S3.** Parameter values selected for simulation G - T belonging to case study-3 comparison. All simulations are based on a year (365 days) with a different value for either the culling capacity or the origin of *M. viscosa/S. parauberis* outbreak. The simulation G, H, I, J, K, L, and M were carried out with the assumption of the first disease outbreak from a different BMA site without any culling strategy (FALSE), but the simulation N, O, P, Q, R, S, and T were simulated with the full culling capacity (135000 fish per day). Except for the values mentioned in this study, the remaining elements were set to their default value.
