# Supplementary figures and images for: Assessments of epidemic spread in aquaculture: comparing different scenarios of infectious bacteria incursion through spatiotemporal hybrid modeling
Source: Front Vet Sci. 2023 Sep 13;10:1205506. doi: 10.3389/fvets.2023.1205506 (PMC10527373; doi:10.3389/fvets.2023.1205506)

## Slide 1
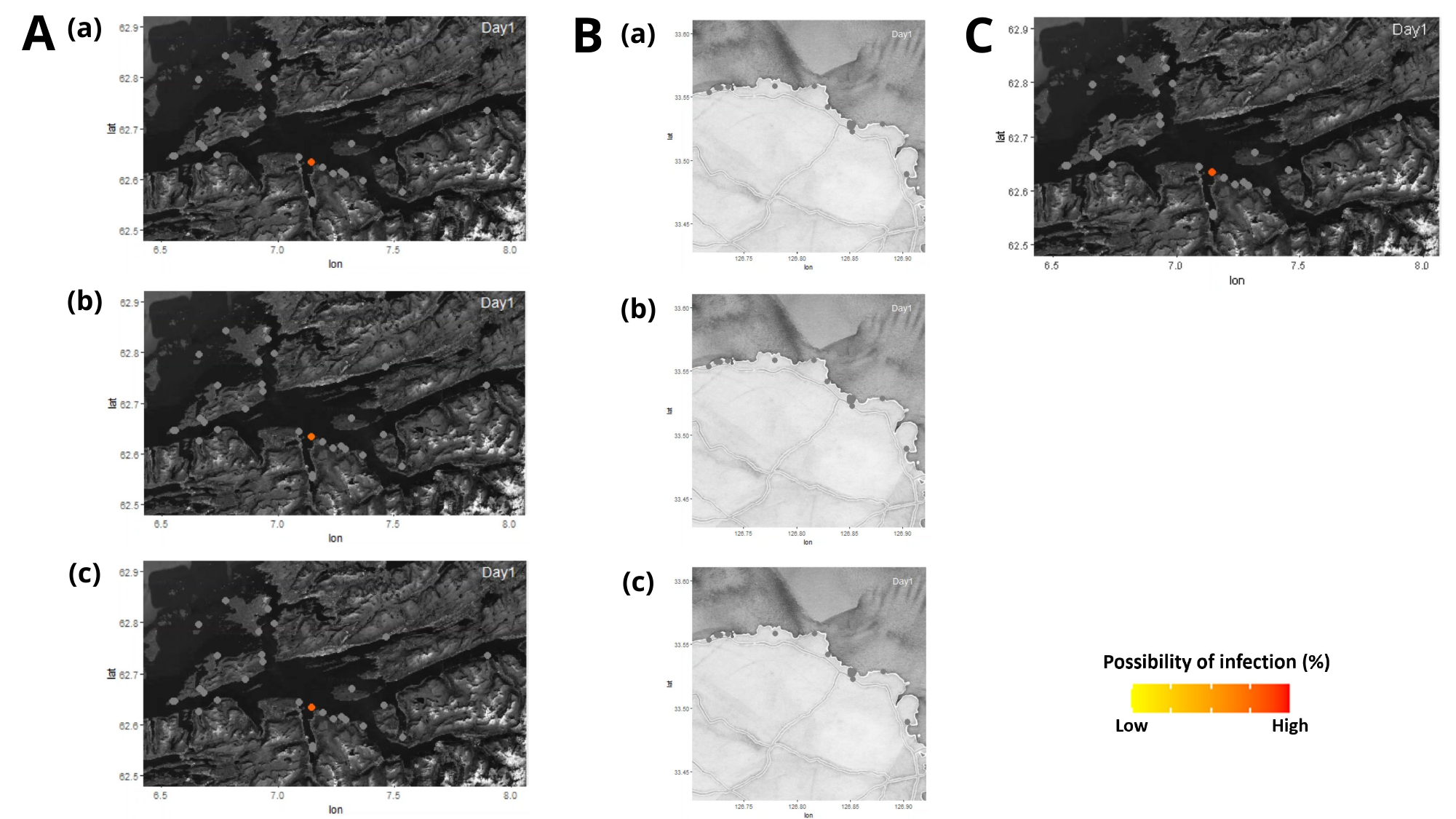

A
(a)
(b)
(c)
(a)
B
(b)
(c)
C

Supplement: Supplementary file 2 [file Presentation_1.PPTX]

## Slide 1
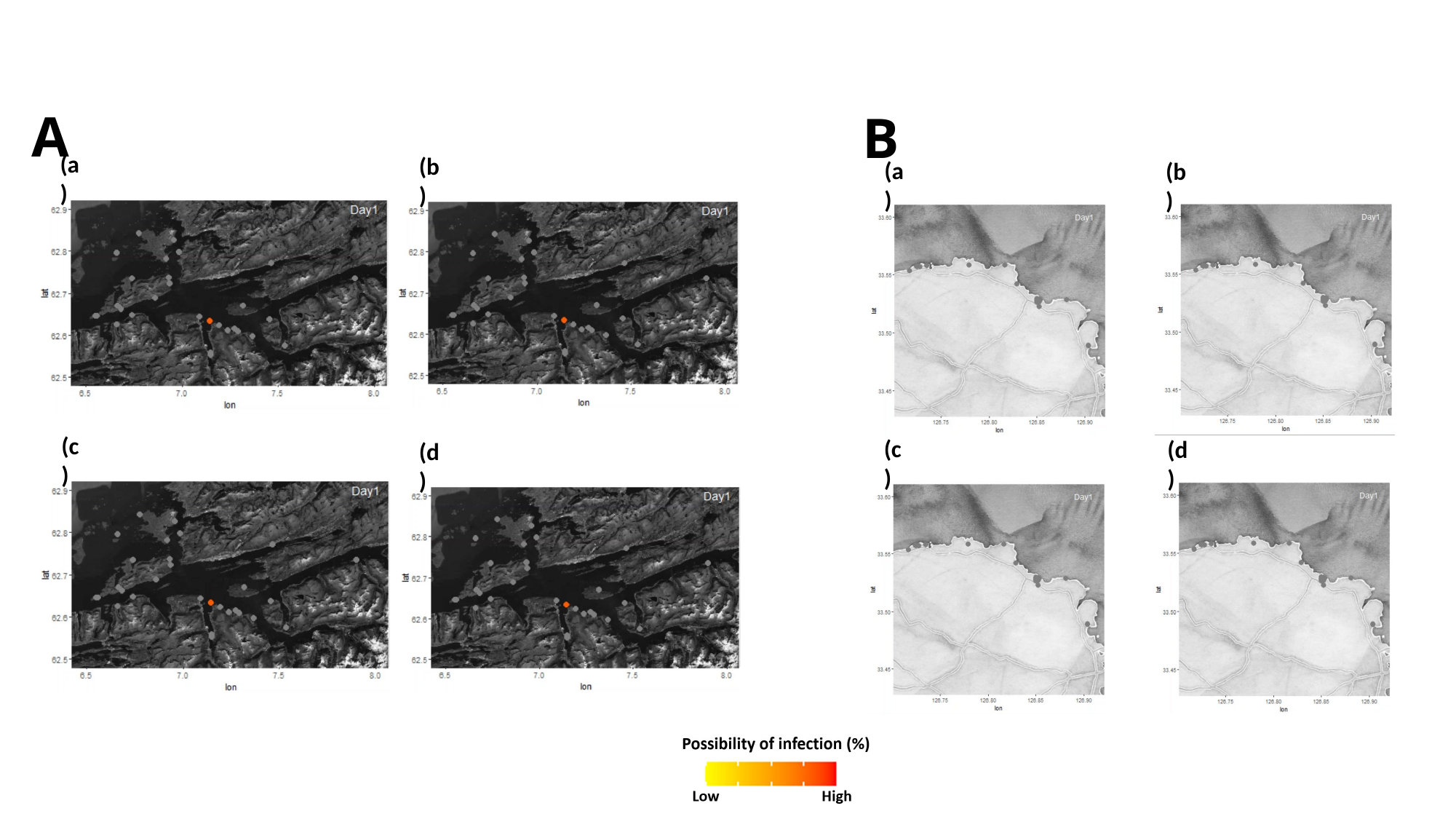

A
B
(a)
(b)
(c)
(d)
(a)
(b)
(c)
(d)

Supplement: Supplementary file 3 [file Presentation_2.PPTX]

## Slide 1
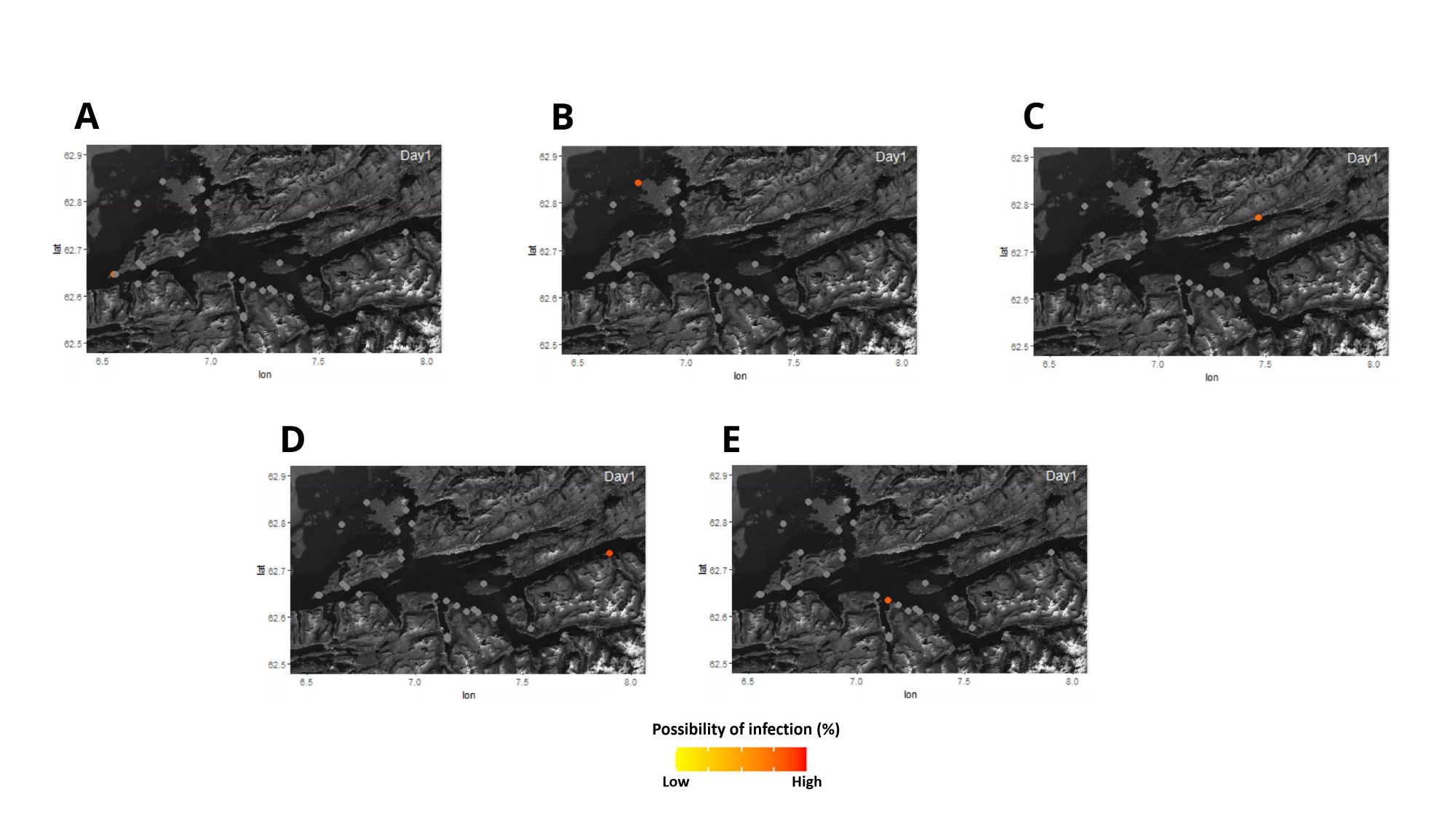

A
C
B
D
E

Supplement: Supplementary file 4 [file Presentation_3.PPTX]

## Slide 1
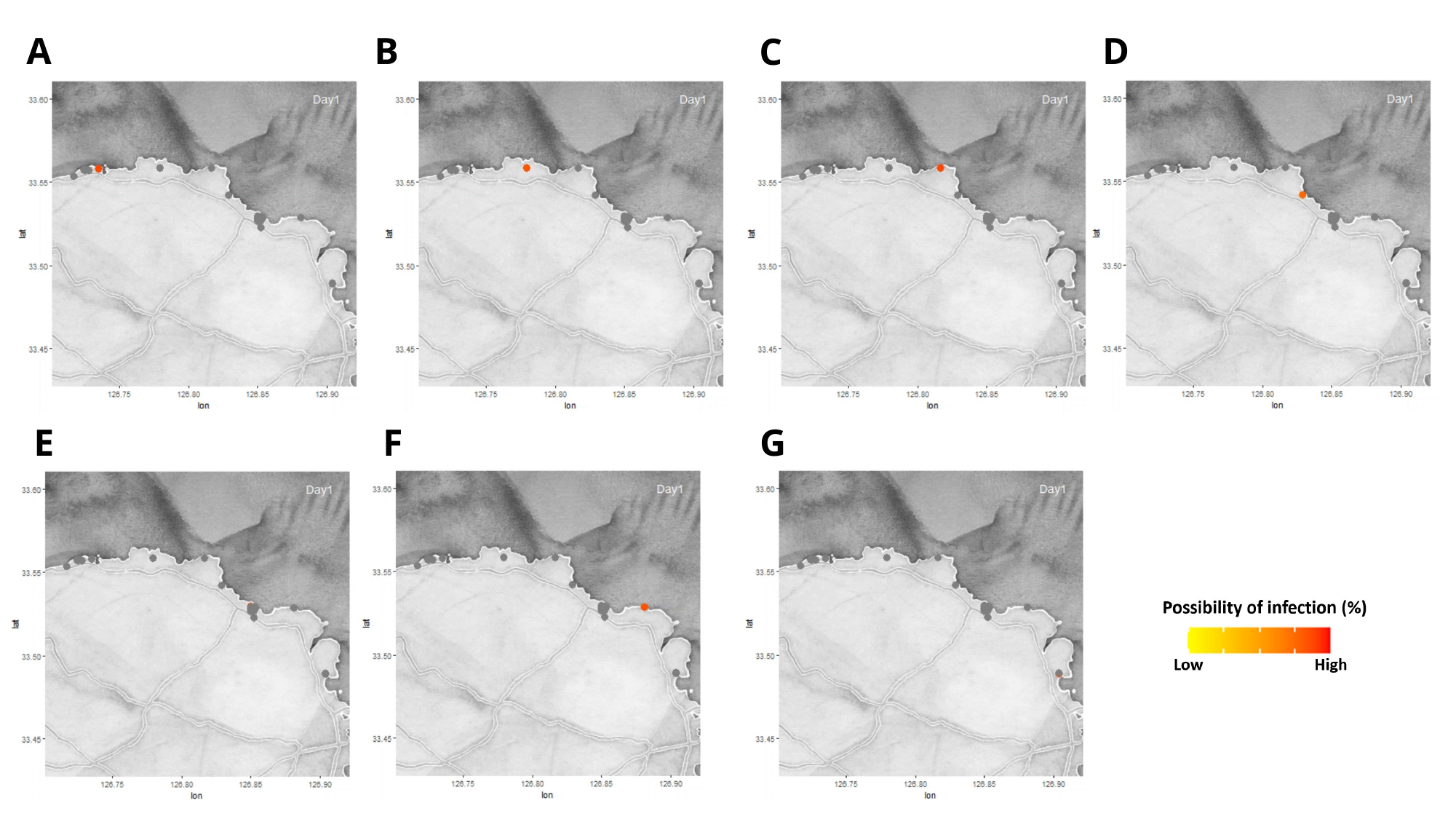

A
B
D
C
E
F
G

Supplement: Supplementary file 5 [file Presentation_4.PPTX]
